# Near‐infrared spectroscopy for the inline classification and characterization of fruit juices for a product‐customized flash pasteurization

**DOI:** 10.1002/fsn3.2709

**Published:** 2022-01-05

**Authors:** Imke Weishaupt, Peter Neubauer, Jan Schneider

**Affiliations:** ^1^ Institute for Life Science Technologies ILT.NRW Department of Life Science Technologies OWL University of Applied Sciences and Arts Lemgo Germany; ^2^ Bioprocess Engineering Department of Biotechnology Technische Universität Berlin Berlin Germany

**Keywords:** flash pasteurization, fruit juice characterization and classification, inline near‐infrared spectroscopy, multivariate data analysis

## Abstract

The feasibility of inline classification and characterization of seven fruit juice varieties was investigated by the application of near‐infrared spectroscopy (NIRS) combined with chemometrics. The findings are intended to be used to optimize the flash pasteurization of liquid foods. More precise information of the kind of product in real time had to be achieved to enable a more product‐specific process. Using the method of partial least squares discriminant analysis, the fruit juice varieties were classified, showing a classification rate of 100% regarding an internal and 69% regarding an external test sets. A characterization by the extract content, pH value, turbidity, and viscosity was made by fitting a partial least squares regression model. The percentage prediction error of the pH value was <3% for internal and external test sets, and for the Brix value prediction errors were about 4% (internal) and 20% (external). The parameters viscosity and turbidity were found to be unsuitable. Despite this, the strategy applied to gain more product‐specific information in real time showed to be feasible. By linking the results to a database containing potentially harmful microorganisms for various types of fruit juices, a more product‐specific calculation of the necessary heat input can be performed. To demonstrate the practical relevance, a comparison between conventional and product‐adapted process control was performed using two fruit varieties as examples in case of *Alicyclobacillus acidoterrestris*. Thus, with more accurate product information, achieved through the use of NIRS with chemometrics, a more precise calculation of the heat input can be achieved.

## Graphical abstract

The feasibility of inline classification and characterization of seven fruit juice varieties was investigated by the application of near‐infrared spectroscopy combined with the chemometric methods of partial least squares discriminant analysis and partial least squares regression. The findings are intended to be used to optimize the flash pasteurization process, where more precise information about the product in real time was necessary. The feasibility of the classification was demonstrated by high classification rates on internal and external test sets, and characterization was enabled using the pH and Brix values.

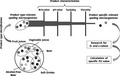

## 1 INTRODUCTION

### 1.1 Motivation

Fruit juices consist of a large number of nutritive compounds like organic acids, amino acids, minerals, vitamins, and in a small amount phytochemicals (Belitz et al., 2009). Preserving this benefit challenges manufacturers to find a compromise between microbiological stability and sensory and nutritional quality. The greatest influence in this sense is exerted by the production step of preservation. In spite of novel techniques, the most common way of preservation remains thermal treatment in terms of high temperature short time (HTST) or bottle pasteurization. The thermal effect required to prevent spoilage by microorganisms is expressed in pasteurization units (PU). The PU are calculated using a highly simplified model (Heiss, 2004; Rahman, 2007). The so‐called fruit juice formula was historically developed based on empirical values (Oliver‐Daumen, 2011; Schwarzer et al., 2010). Even if dedicated particularly to fruit juices, simplification of this formula provides insufficiencies, which can lead to both impaired safety and exaggerated treatment. The major disadvantage of the PU model results first from “globalization” by employing a product‐unspecific leading germ as reference and second from ignoring the heating and cooling sections in a continuous flash pasteurization system. For a realistic assessment of the microbiological hazard potential, product properties such as pH value, extract content (“Brix value”), and turbidity have to be taken into account. These parameters can affect the inactivation rate of microorganisms, which is expressed by *D* and *z* values. The *D* value is the decimal reduction time at a reference temperature. The *z* value indicates the required temperature increase that is necessary to reduce the *D* value (time) to a tenth compared to a reference temperature (Tiwari & Rajauria, 2018). Depending on the fruit juice type and its individual properties, the *D* and *z* values for the microorganisms vary in a relevant extent (Oliver‐Daumen, 2011). Therefore, getting information about the product in time of processing enables to determine more product‐specific *D* and *z* values, provided that a database with corresponding values like Brix and pH values can be accessed. Using this information, a more specific PU target value can be calculated and the process control can be adjusted accordingly (Schwarzer et al., 2010). Such a database with scientifically approved *D* and *z* values for specific product type and the specific growth conditions already exists with an open Internet access and is continuously fed with new data (Schwarzer et al., 2010). Hence, the aim of this study is to enable manufacturers to profit practically from this database, to obtain a more gentle process, and to better protect nutritive juice compounds. Therefore, the feasibility of inline product classification (fruit variety) and characterization of relevant properties (Brix value, turbidity, pH value, and viscosity) was demonstrated and presented in this study.

### 1.2 Approach for a case‐specific pasteurization

**FIGURE 1:**
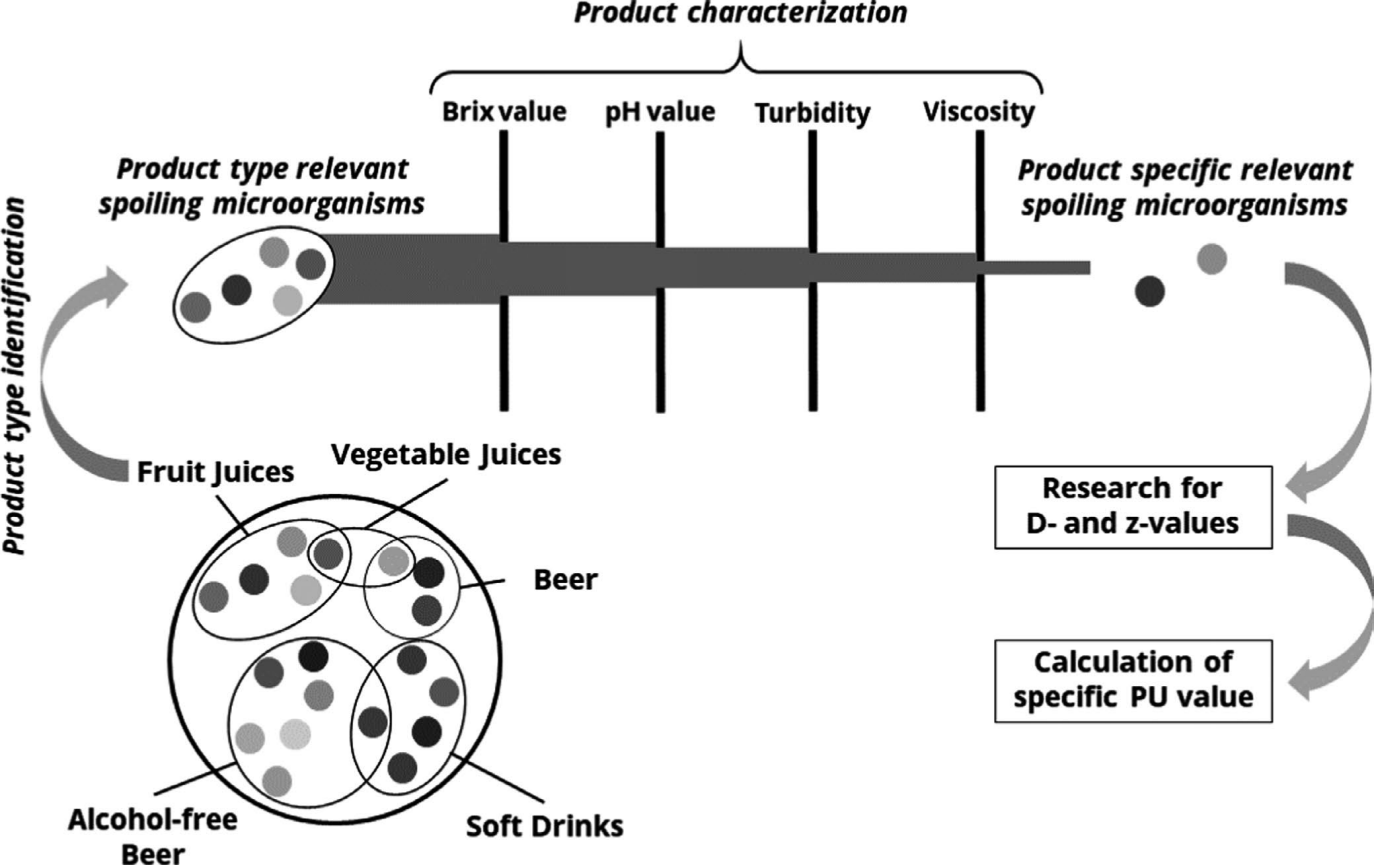
Underlying background idea as a novel approach for a case‐specific calculation of the pasteurization units (PU) by the individual selection of spoilage microorganisms from fruit variety identification and analysis of relevant product properties

This work follows a novel approach to liquid food preservation that allows for case‐specific pasteurization and better protects valuable chemical compounds. Figure 1 shows the underlying idea and workflow, which is investigated here using near‐infrared (NIR) analysis. With the help of inline spectroscopy measurements and chemometrics, a selection of the microorganisms that are specifically relevant for the product is to be made from a large number of potentially harmful microorganisms. Only for these microorganisms, the kinetic parameters (*D*/*z* values) have to be considered, with which a (“pessimistic”) PU calculation is performed. Although these resulting PU are still to be regarded as a worst‐case scenario for safety reasons, this only refers to the ultimately selected few microorganisms. This calculation is therefore much more optimistic and thus gentler than the globalizing assumption of a much larger spectrum of microorganisms. The selection of harmful microorganisms in practical applications is supposed to be conducted in two steps: (i) the categorization of the product type and variety and (ii) the product characterization with regard to Brix, pH, turbidity, and viscosity. The aforementioned strategy is exemplified within this study using two fruit varieties (apple and grape; of a total of seven varieties contained in this study).

### 1.3 Near‐infrared spectroscopy application on fruit juices

For the determination of product information, a real‐time and inline applicable analytical method, which is nondestructive and easily adaptable, is necessary. Near‐infrared spectroscopy (NIRS) in combination with chemometrics has the potential to accomplish these requirements (Günzler & Gremlich, 2002; Kessler, 2007). Near‐infrared spectroscopic investigations on fruit juices are the matter of numerous publications. Typical applications are the verification of the correct declaration of regional provenance, the verification of authenticity, or the quantification of special ingredients (Hosseini et al., 2021; Igual et al., 2010; Kelly & Downey, 2005; Lanza & Li, 1984; Rambla et al., 1997; Reid et al., 2005; Šnurkovič, 2013; Twomey et al., 1995; Włodarska et al., 2018). In most cases, these investigations are offline applications and place emphasis on a single fruit variety or on particular ingredients. Some studies combine different techniques such as NIRS with ICA like in the study of Ribeiro et al. (2017). An example of a study involving a multiproduct investigation was published by dos Santos et al. (2018). Beside the specific aim in the context of the fruit juice pasteurization and NIRS as an inline measurement method, a particularity of this work is the use of a so‐called transflection probe, which is a system of transmission measurement with a doubled path length using a reflection surface.

## 2 MATERIALS AND METHODS

**FIGURE 2:**
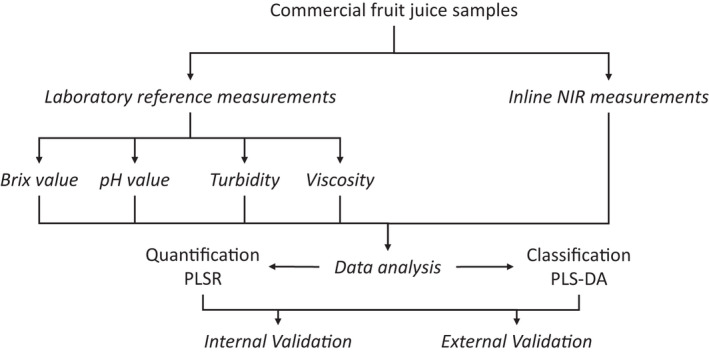
Scheme of the experimental design

Figure 2 shows schematically the experimental design of processing and the ways of analyzing and evaluating the results by chemometric methods, which is explained in more detail in the following sections.

### 2.1 Sample material

In order to study the feasibility of the classification and quantification of fruit juices by NIRS, 7 × 5 (*n* = 35) commercial samples have been bought from local grocery stores: seven varieties of fruit juices, each from five different producers. The samples included the varieties cloudy apple, orange, pear, peach, cranberry, black currant, and grape. The reference values determined for the parameters investigated within this study are tabulated as mean values with related standard deviation in Table 1. The minimum and maximum values were presented in Table 2. In addition, 26 other fruit juices of different varieties were purchased, the number varying depending on the availability of different manufacturers.

**TABLE 1:** Reference values of *n* = 35 commercially purchased juices

Sample no.	Apple	Pear	Peach	Orange	Grape	Cranberry	Black currant
Brix value (°Brix)							
1	11.56 ± 0.04	12.66 ± 0.02	12.47 ± 0	10.97 ± 0	15.93 ± 0	11.22 ± 0.02	13.89 ± 0
2	10.86 ± 0.03	13.75 ± 0.02	12.05 ± 0.03	10.70 ± 0.02	16.19 ± 0	10.25 ± 0.0	9.86 ± 0
3	11.67 ± 0	12.97 ± 0	12.46 ± 0.09	11.00 ± 0.01	16.15 ± 0	11.24 ± 0.0	12.68 ± 0.02
4	11.54 ± 0	12.19 ± 0	14.63 ± 0.02	11.12 ± 0.01	16.51 ± 0	11.68 ± 0	12.31 ± 0
5	12.00 ± 0	12.69 ± 0.01	13.11 ± 0.02	11.02 ± 0.01	16.15 ± 0	12.79 ± 0.02	10.19 ± 0
pH value (–)							
1	3.52 ± 0.01	3.65 ± 0.03	3.58 ± 0.01	4.1 ± 0.01	3.44 ± 0.01	2.85 ± 0.02	2.92 ± 0.04
2	3.59 ± 0.01	3.71 ± 0.01	3.58 ± 0	3.91 ± 0	3.42 ± 0.02	2.85 ± 0.01	2.95 ± 0.02
3	3.44 ± 0	3.53 ± 0.01	3.61 ± 0.01	3.92 ± 0	3.41 ± 0	2.77 ± 0.01	2.95 ± 0
4	3.32 ± 0.01	3.70 ± 0.01	3.68 ± 0.01	3.87 ± 0.01	3.32 ± 0.01	2.75 ± 0.01	2.96 ± 0.01
5	3.45 ± 0.01	3.49 ± 0.01	3.51 ± 0.01	3.87 ± 0	3.42 ± 0	2.59 ± 0.01	2.92 ± 0.01
Turbidity (EBC)							
1	131 ± 0	922.6 ± 2.6	1068.4 ± 20.7	983.2 ± 0.4	0.5 ± 0	197.8 ± 0.8	0.1 ± 0
2	242.6 ± 0.5	226.6 ± 0.9	1039.6 ± 6.6	1045 ± 0	0.5 ± 0	1.4 ± 0	0.7 ± 0
3	324 ± 0	596.6 ± 1.7	980.2 ± 2.6	1184.2 ± 0.4	0.2 ± 0	1.3 ± 0	1.4 ± 0
4	166.8 ± 0.4	75.8 ± 0.4	738.6 ± 11.8	1357.8 ± 0.4	0.2 ± 0	3.1 ± 0	0.5 ± 0
5	275.4 ± 0.5	824.6 ± 3.8	592 ± 0.7	1502.2 ± 1.6	0.3 ± 0	6.2 ± 0	0.3 ± 0
Viscosity (mPas)							
1	1.11 ± 0.18	20.64 ± 1.25	13.68 ± 0.59	2.29 ± 0.34	2.43 ± 0.09	1.33 ± 0.15	2.36 ± 0.08
2	1.27 ± 0.02	4.12 ± 0.29	13.87 ± 0.8	3.38 ± 0.18	2.12 ± 0.18	1.02 ± 0.05	2.22 ± 0.04
3	0.92 ± 0.07	10.8 ± 0.54	9.83 ± 0.88	2.68 ± 0.08	2.00 ± 0.15	1.03 ± 0.04	2.03 ± 0.13
4	1.79 ± 0.04	2.06 ± 0.06	17.74 ± 0.25	2.50 ± 0.1	2.03 ± 0.07	1.03 ± 0.02	1.92 ± 0.25
5	1.43 ± 0	11.42 ± 0.9	10.73 ± 0.57	3.76 ± 0.08	2.51 ± 0.14	1.11 ± 0.03	2.29 ± 0.04

Note

Values are mean ± standard deviation of Brix and pH values, viscosity, and turbidity examined in a fivefold measurement.

**TABLE 2:** Minimum (top of cell) and maximum (bottom of cell) values of the fivefold measurements for the reference values of *n* = 35 commercially purchased juices; examined were values of Brix and pH values, viscosity, and turbidity

Sample no.	Apple	Pear	Peach	Orange	Grape	Cranberry	Black currant
Brix value (°Brix)							
1	11.53	12.65	12.47	10.97	15.9	11.20	13.89
	11.60	12.70	12.47	10.98	15.93	11.24	13.89
2	10.81	13.74	12.03	10.66	16.19	10.25	9.86
	10.88	13.77	12.08	10.71	16.19	10.25	9.86
3	11.67	12.97	12.38	10.99	16.15	11.24	12.66
	11.67	12.97	12.57	11.00	16.15	11.24	12.70
4	11.54	12.19	14.61	11.11	16.51	11.68	12.31
	11.54	12.19	14.65	11.14	16.51	11.68	12.31
5	12.00	12.69	13.09	11.00	16.15	12.76	10.19
	12.00	12.70	13.13	11.03	16.15	12.81	10.19
pH value (–)							
1	3.52	3.60	3.57	4.10	3.44	2.82	2.88
	3.53	3.67	3.58	4.11	3.45	2.88	2.97
2	3.57	3.70	3.58	3.90	3.39	2.84	2.92
	3.60	3.73	3.59	3.91	3.43	2.87	2.96
3	3.44	3.53	3.60	3.92	3.40	2.76	2.95
	3.45	3.54	3.62	3.92	3.41	2.79	2.95
4	3.32	3.69	3.67	3.86	3.32	2.74	2.95
	3.33	3.70	3.69	3.88	3.33	2.76	2.98
5	3.45	3.48	3.50	3.87	3.41	2.58	2.91
	3.46	3.50	3.51	3.87	3.42	2.60	2.93
Turbidity (EBC)							
1	131	919	1043	983	0.49	197	0.08
	131	925	1091	984	0.52	199	0.09
2	242	226	1032	1045	0.43	1.43	0.74
	243	228	1049	1045	0.52	1.47	0.76
3	324	594	976	1184	0.22	1.28	1.36
	324	598	983	1185	0.24	1.30	1.41
4	166	75	726	1357	0.16	3.08	0.48
	167	76	754	1358	0.21	3.15	0.57
5	275	821	591	1500	0.28	6.17	0.24
	276	830	593	1504	0.33	6.26	0.30
Viscosity (mPas)							
1	0.84	19.13	12.80	1.83	2.32	1.14	2.23
	1.24	22.30	14.24	2.67	2.57	1.48	2.43
2	1.24	3.71	12.95	3.16	1.98	0.98	2.18
	1.29	4.45	14.63	3.56	2.42	1.09	2.27
3	0.84	9.89	8.85	2.57	1.88	0.99	1.93
	1.04	11.32	10.58	2.78	2.23	1.09	2.18
4	1.73	1.98	17.40	2.37	1.93	0.99	1.73
	1.83	2.13	18.04	2.62	2.08	1.04	2.32
5	1.43	10.63	10.04	3.65	2.27	1.09	2.24
	1.43	12.90	11.37	3.86	2.62	1.14	2.32

### 2.2 Inline NIR measurements

Processing of fruit juice samples and near‐infrared inline measurements were carried out in a laboratory system for HTST treatment of liquid foodstuff type HT220 (OMVE). The experimental setup has been described in detail previously (Weishaupt et al., 2020) so that only a brief description of the general setup is given here. The inline NIR measurements were conducted under constant process conditions with flow rate of 90 L/h, temperature of 20°C, and pressure of 3 bar. The heat‐holding section of the HTST laboratory plant was extended by a tube coil containing a segment with three ports that allows the insertion of external probes. Through one of these ports the NIR probe was inserted. The NIR probe is a so‐called transflection probe sensor with a variable path length and a reflective surface at the opposite side of the light source (Avantes BV). The sensor was connected to a spectrometer type PSS‐2120 (Polytec GmbH) with a diode array detector of 256 pixels and a spectral range from 1100 to 2100 nm in combination with the software Pas Labs 1.2 (Polytec GmbH). Recording of the spectra was made in absorbance mode with 100 scans averaged per spectrum. The path length of transflection probe was set to 2 mm. Before running the fruit juice samples, a reference measurement with demineralized water at a temperature of 20°C was conducted, also under constant process conditions with flow rate of 90 L/h. Each juice was measured inline 30 times at 20°C. For the generation of a so‐called external test set of spectral data, which are totally independent from the training dataset, another set of 26 different commercial fruit juices consisting of the same varieties were measured under the same process settings.

### 2.3 Laboratory reference measurements

Extract (“Brix value”), turbidity, pH value, and viscosity were measured in the laboratory environment to provide reference data, which were used to generate models using the NIR spectra with the help of chemometric methods. These reference values are necessary in the second step after identification of the fruit variety to better individualize the pasteurization requirements according to the overall approach described earlier (Figure 1). The Brix value was measured with a refractometer type J157 Automatic Refractometer (Rudolph Research Analytical, Hackettstown, NJ) by placing a small amount of sample in the measuring chamber. The turbidity was measured with the laboratory turbidity meter type 2100AN (Hach) by filling a measuring cuvette with sample material and placing it in the measuring cell. The turbidity meter features a triple beam scattered light optical design (590–1100 nm wavelength), measuring a side (90° angle) scatter of the sample. The pH value was measured with a pH meter Protos 3400S (Knick Elektronische Messgeräte GmbH & Co. KG). The viscosity measurements were carried out at 20°C with a rotational viscometer ViscoQC 300 (Anton Paar Germany GmbH, Ostfildern‐Scharnhausen, Germany) with the adapter CC12 and 230 rpm for 1 min measuring time. All measurements were performed in fivefold, for which the mean and standard deviation were then calculated.

### 2.4 Data analysis

The characterization and classification of the fruit juices were realized with chemometric methods applying Simca 16.1 (MKS Umetrics AB). An informative overview of the basics of the chemometric methods used here is given by Hosseini et al. (2021) in their publication, thus following is a brief explanation of the partial least squares regression (PLSR) and partial least squares discriminant analysis (PLS‐DA) methods used here. For characterization through quantification of ingredients, the PLSR method was applied. The PLSR is the regression extension of the principal component analysis (PCA), in which a dimension reduction takes place with simultaneous correlation to the results of reference analytics. This is achieved by simultaneously modeling the spectra of the fruit juice samples (Matrix **X**) and the values of the reference analytics (Matrix **Y**), looking for the latent variables in **X** that are most predictive of those in **Y**, respectively. The overall aim of PLSR is to predict properties of unknown samples (Eriksson, 2013). PLSR models for Brix value, turbidity, viscosity, and pH value were fitted and validated on an internal test set (spectral dataset was split into 2/3 training and 1/3 test set) and on an external test set (26 totally independent fruit juice samples). For classification, the method of PLS‐DA was employed, which is an adaption of the PLSR for the purpose of classification (Aguilar‐Rosas et al., 2007; Ruiz‐Perez et al., 2020; Szymańska et al., 2012). The principle is based on the use of a binary “dummy” system, which assigns, for example, a 0 or a 1 depending on the class membership. With a chosen threshold of 0.5, a sample is considered to be a group member, when the predicted *y* value is the highest and it has a value above 0.5. For the ability of allocation, the model has to be trained in a calibration phase regarding the characteristics of the individual groups. In this study, the aim of the categorization was to distinguish the seven different fruit varieties. Before the regression and classification models were developed, the raw spectra were preprocessed. For this matter, regardless of the chemometric method applied, the dataset of 30 spectra recorded for each fruit juice sample was divided into two parts, 20 spectra as training set and 10 spectra as internal test set. With tools like wavelength selection, standard normal variation (SNV), multiscatter correction (MSC), Savitzky–Golay smoothing, and derivative spectra, model performance was optimized in an iterative process trying to find the best preprocessing strategy for a high‐performing prediction or classification model. Quality parameters of evaluation were the explained variation (*R^2^*) and the predictive quality (*Q* ^2^). *R* ^2^ represents a measure for the variance explained by the model and *Q* ^2^ for the predictive ability based on the difference between the predicted value and the actual value. Both shall attain a value close to 1 as indication of a high performance. The number of latent variables formed in the course of dimensional reduction was examined to avoid overfitting by executing the permutation test (Eriksson, 2013; Lindgren et al., 1996). This has the advantage of checking the optimal number of latent variables and of verifying the statistical significance of the model. Starting from the unpermuted model with corresponding *R* ^2^ and *Q* ^2^, the Y variable is randomly mixed up in its assignment to the X variable. This leads then to changed values of *R* ^2^ and *Q* ^2^. If the original model is high in significance, the *R* ^2^ and *Q* ^2^ resulting from the permutation test are supposed to be significantly lower (*R* ^2^ below 0.3 and *Q* ^2^ lower than 0.05; Eriksson, 2013; Eriksson et al., 2008; Lindgren et al., 1996).

In addition to these general quality parameters, there are further quality and performance parameters specific for PLSR and PLS‐DA. For PLSR, the root mean square error of cross‐validation (RMSECV) and the root mean square error of estimation (RMSEE) were calculated to evaluate the regression model quality. The RMSECV is a measure for the prediction error in case of internal cross‐validation and the RMSEE is calculated by the comparison of given and estimated values of Y variable. Both should be low and as close to each other as possible. The root mean square error of prediction (RMSEP) results from an internal validation using the internal test set. It represents the model performance regarding the prediction of unknown samples. To proof the model performance, the RMSEP for an external test set was also calculated, which contains only independent fruit juice samples.

In order to assess the classification performance of PLS‐DA models, there are – besides the overall percentage of correct classification – several parameters, which specifically describe the classification performance better. Among these, the most common ones are the sensitivity (*se*) and the specificity (*sp*). The sensitivity describes the proportion of true positive assignments (*TP*) to the total number of class members consisting of true positive and false negative (*FN*) results. The specificity describes the ratio between true negative (*TN*) class assignments to the total number of members, which are not part of the class under consideration (sum of true negative and false positive [*FP*] assignments). For the calculation of these, the formulas $se=\frac{\mathrm{TP}}{TP+FN}$ and $sp=\frac{\mathrm{TN}}{TN+FP}$ were used. Both have to be near 1 for a high classification performance, whereas *se* and *sp* are inversely proportional (Brereton, 2018; Cozzolino et al., 2012; Oliveri & Downey, 2012; Szymańska et al., 2012). Thus, for a more general statement about the number of correct classifications, the two parameters are combined in the accuracy (*acc*), which is a measure of the degree of truthfulness of the classification. It can be calculated with $acc=\frac{TP+TN}{TP+TN+FP+FN}$ (Oliveri & Downey, 2012). In case of a multiclassification situation with threshold, it is necessary to visualize the classification performance at various thresholds to find the optimum ratio of *se* to *sp* for high performance. For this purpose, the receiver operator characteristic (ROC) plot is considered, in which the sensitivity as true positive rate (*TPR*) is plotted against the false positive rate (*FPR* = 1 − *sp*). In case of more than two classes, it represents the separability of one group against all other groups. This curve should therefore rise as steeply as possible (ideal case would be *TPR* = 1 and *FPR* = 0). If the curve runs close to the bisector, a purely coincidental allocation can be assumed. The threshold value that has the largest normal distance from the bisector is the optimal one. As a measure for the curse of ROC curve, the area under the curve (*AUC*) was introduced, which has a value of 0.5 for the bisector. The closer this value comes to 1, the better the ability to separate between different classes is. The AUC value thus corresponds to the probability that a positive value is actually classified as such (Baratloo et al., 2015; Fawcett, 2006; Oliveri & Downey, 2012; Szymańska et al., 2012).

### 2.5 Scenario of a smart selection of pasteurization parameter setting using product‐specific *D* and *z* values

For a more product‐specific control of the pasteurization process, information about the product to be pasteurized is necessary, such as the fruit variety, the Brix value, and the pH value. Within this study, this information is determined by NIRS and chemometric methods. Considering two fruit juices, for example, apple and grape are processed in one production site. First, they need to be identified as apple juice or grape juice by NIR and PLS‐DA. Then, Brix and pH values are predicted using NIR measurement and PLSR. After determining these specific microbiologically relevant parameters by means of NIR, product‐specific characteristic data of the mortality rates (*D* and *z* values) could be determined from the database. Via comparison with the globalized PU values determined by applying fruit juice formula, the extent of a possible optimization by a more product‐specific calculation of the PU values.

## 3 RESULTS AND DISCUSSION

The foundation for a more product‐specific pasteurization is the knowledge of certain product properties, which must already be available at the start of production. The microbially relevant properties to be determined are the fruit variety, the extract content, the pH value, the turbidity, and the viscosity, which are realized by means of NIR spectra in combination with chemometrics. The aim of the following data evaluation is therefore to test the feasibility of this approach.

### 3.1 Identification of fruit variety

**FIGURE 3:**
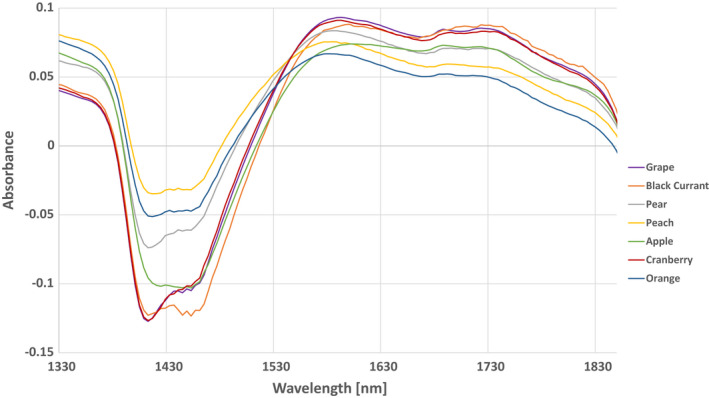
Typical spectra after preprocessing for each fruit variety examined

Near‐infrared raw spectra (1050 measurements) were divided into a training set (700 measurements), that is, 20 spectra per fruit juice, and a test set (350 measurements), that is, 10 spectra per fruit juice. Two models were generated, one for fruit variety classification applying PLS‐DA and another one for characterization of fruit juice properties applying PLSR. For both models, the preprocessing methods of wavelength selection and MSC led to highest model quality parameters, relying on the parameters of *R* ^2^ and *Q* ^2^ (data shown in Table 3). Besides the parameters *R* ^2^ and *Q* ^2^, it was mainly the prediction performance expressed in RMSEP with respect to the external test set, since low prediction error values are associated with a high robustness of the model against unknown samples. A comparison of all the results of the different preprocessing methods is shown in Table 4 for the PLS‐DA model and in Table 5 for the PLS model. With a number of 12 latent variables, the permutation test resulted in very low values in case of PLS‐DA model, indicating a statistical significance and no overfitting, and with a number of 11 latent variables for PLSR model, respectively. Although the permutation test induced that it is not overfitting, the number of LVs is high considering the number of samples used. To reduce the risk of overfitting, pseudo‐univariate models could be an alternative. Figure 3 shows an example of spectra for each fruit variety after preprocessing.

**TABLE 3:** Characteristics and quality parameters of PLS‐DA model for discrimination of fruit variety and PLSR model for prediction of fruit juice characteristics

Model type	Preprocessing methods	Number of latent variables (LV)	Wavelength area (nm)	Number of spectra included	Explained variation (*R*_X_^2^/*R*_Y_^2^)	Predictive quality (*Q* ^2^)
PLS‐DA	MSC	12	1332–1441 1465–1837	700	1/0.959	0.958
PLS‐DA orange		8			1/0.967	0.966
PLSR		11			1/0.946	0.944

**TABLE 4:** Comparison of model and prediction quality parameters of PLS‐DA models fitted by using different preprocessing methods and 700 spectra included (LV, latent variables; class. rate, classification rate; 1. der, first derivative; 2. der, second derivative; ext. TS, external test set)

Preprocessing method	Number of LV	Wavelength area (nm)	Explained variation (*R*_X_^2^/*R*_Y_^2^)	Predictive quality (*Q* ^2^)	Class. rate ext. TS (%)
MSC	12	1332–1441 1465–1837	1/0.959	0.958	69
SNV			1/0.961	0.96	65
SG			1/0.941	0.94	46
1. der			1/0.945	0.945	58
2. der			0.989/0.942	0.941	46

**TABLE 5:** Comparison of model and prediction quality parameters of PLS models fitted by using different preprocessing methods with 700 spectra included, 11 latent variables, a wavelength selection from 1332 to 1441 nm and 1465 to 1837 nm (1. der, first derivative; 2. der, second derivative; ext. TS, external test set)

Preprocessing method	Explained variation (*R*_X_^2^/*R*_Y_^2^)	Predictive quality (*Q* ^2^)	RMSEP ext. TS Brix	RMSEP ext. TS pH	RMSEP ext. TS viscosity	RMSEP ext. TS turbidity
MSC	1/0.959	0.958	2.33	0.3	3185.37	940.34
SNV	1/0.961	0.96	3.46	0.35	3184.99	136.55
SG	1/0.941	0.94	2.82	0.3	3179.92	303.39
1. der	1/0.945	0.945	3.16	0.82	3182.52	307.58
2. der	0.989/0.942	0.941	2.42	0.98	3181.4	557.13

After evaluation of model quality, the classification performance of the PLS‐DA model was validated by calculating the sensitivity and specificity, which were merged to get the parameter of accuracy. The classification performance for the calibration dataset and the internal test dataset showed no failures in classification. Regarding the external test dataset which consists of 26 completely independent juices, the classification rate decreased from 100% to 69% (Table 6), which still corresponds to a high assignment rate.

**TABLE 6:** Classification table of PLS‐DA model for the external test set; incorrect classification is marked in bold and italics

Samples	Class members	Correct classified	Classified as						
			Grape	Black currant	Orange	Cranberry	Apple	Pear	Peach
Grape	5	80%	4	0	0	***1***	0	0	0
Black currant	3	33.33%	***1***	1	0	***1***	0	0	0
Orange	4	100%	0	0	4	0	0	0	0
Cranberry	3	100%	0	0	0	3	0	0	0
Apple	3	33.33%	0	0	0	0	1	***2***	0
Pear	5	40%	***1***	0	***2***	0	0	2	0
Peach	3	100%	0	0	0	0	0	0	3
Total	26	69%	6	1	6	5	1	4	3

**FIGURE 4:**
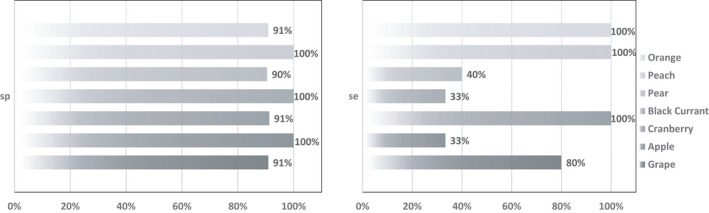
Performance parameter specificity (*sp*) and sensitivity (*se*) of PLS‐DA model calculated with the external test set

Expressed in terms of the performance parameters *se*, *sp*, and *acc*, values >90% are shown for the parameter *sp* for all fruit juices. The risk for a wrong assignment of samples to another class is therefore low. For *se*, the values differ greatly depending on the fruit variety. For orange, peach, and cranberry, *se* is 100%, for grape still 80%. Only apple, pear, and black currant have a lower percentage of 33%–40%. This means a higher risk of being assigned to the wrong fruit variety. Results of the parameters *sp* and *se* are shown in Figure 4. Summarized in the *acc*, values >80% are obtained. With the exception of the varieties pear and grape, all varieties showed values greater than 90%. This further corroborates the values of the classification rate, which suggests a good classification rate, since the *acc* does not only consider the correct classification within a variety, but takes into account the entire sample material, that is, all classes.

**FIGURE 5:**
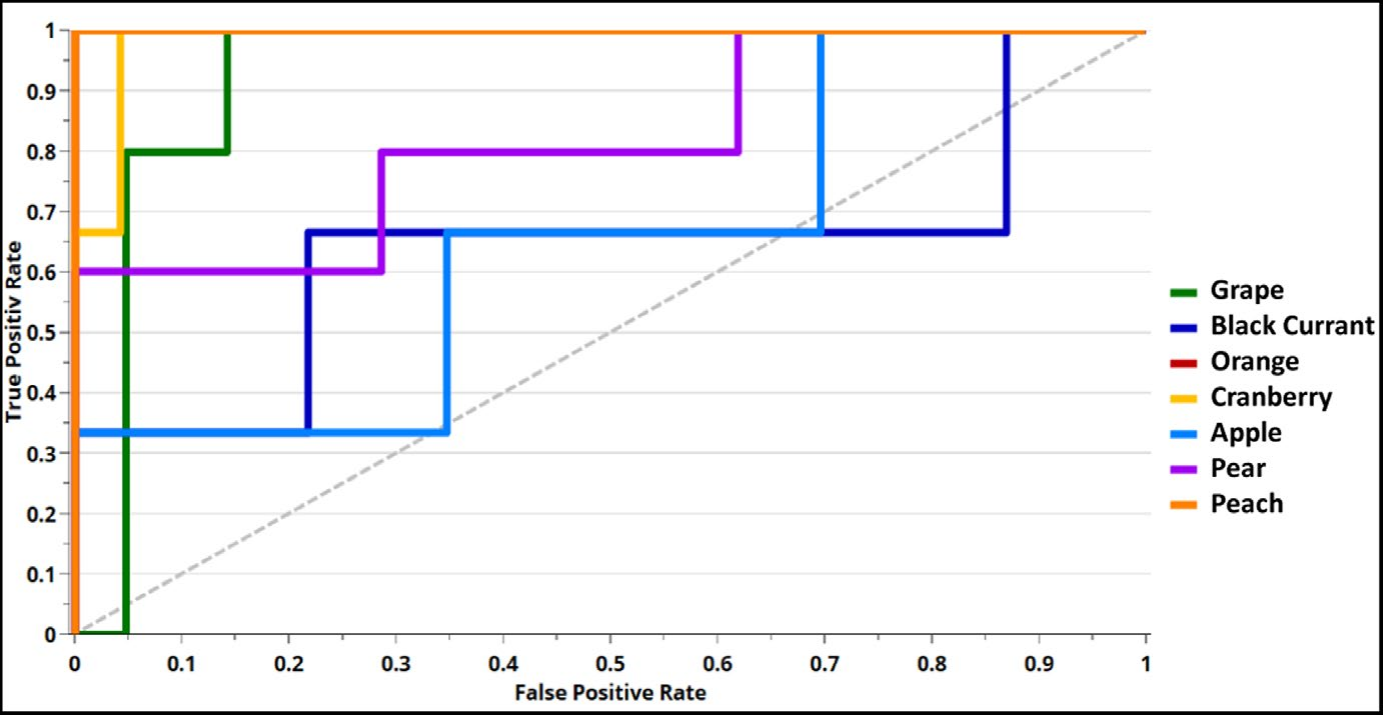
ROC plot of PLS‐DA model with true positive rate (TPR) plotted over false positive rate (FPR) with an optimum value of 1 for TPR and 0 for FPR representing high classification performance; colored lines represent the TPR of the corresponding class at the different thresholds

For a more class‐specific evaluation of performance with different threshold values of the assignment, the ROC curves were plotted (Figure 5) and AUC values were calculated. The different courses of the ROC curves demonstrated the variation in optimal threshold for a high classification performance of the various fruit juices. In the case of grape, orange, cranberry, and peach, the AUC value is between 0.9 and 1, which indicates a high probability for a true positive classification. Pear also has a high AUC value of 0.82. Solely, black currant and apple have values around 0.65, which implies that the selection of a high threshold entails a risk of incorrect classification. Results of AUC calculation are shown in Table 7. To investigate an optimization approach to the model performance, the classification task was modified in a second PLS‐DA model (model properties were displayed in Table 3 named “PLS‐DA orange”). The task was now not to classify each fruit variety used in the study, but to distinguish one variety, in this case orange, from the others. This approach increased the classification rate from 67% to 98%, while the sensitivity for orange remained at 100% and was increased to 95% in the case of specificity. Based on the overall high values of performance parameters, especially since the risk of a false negative assignment is higher than the opposite, the NIRS can be considered as applicable as inline analysis method for classification of fruit juice varieties. Furthermore, it became apparent that there is even potential for improvement.

**TABLE 7:** Classification performance of PLS‐DA model expressed as AUC value regarding the external test set

Fruit variety	AUC value
Grape	0.93
Black currant	0.64
Orange	1
Cranberry	0.99
Apple	0.65
Pear	0.82
Peach	1

### 3.2 Product characterization

**FIGURE 6:**
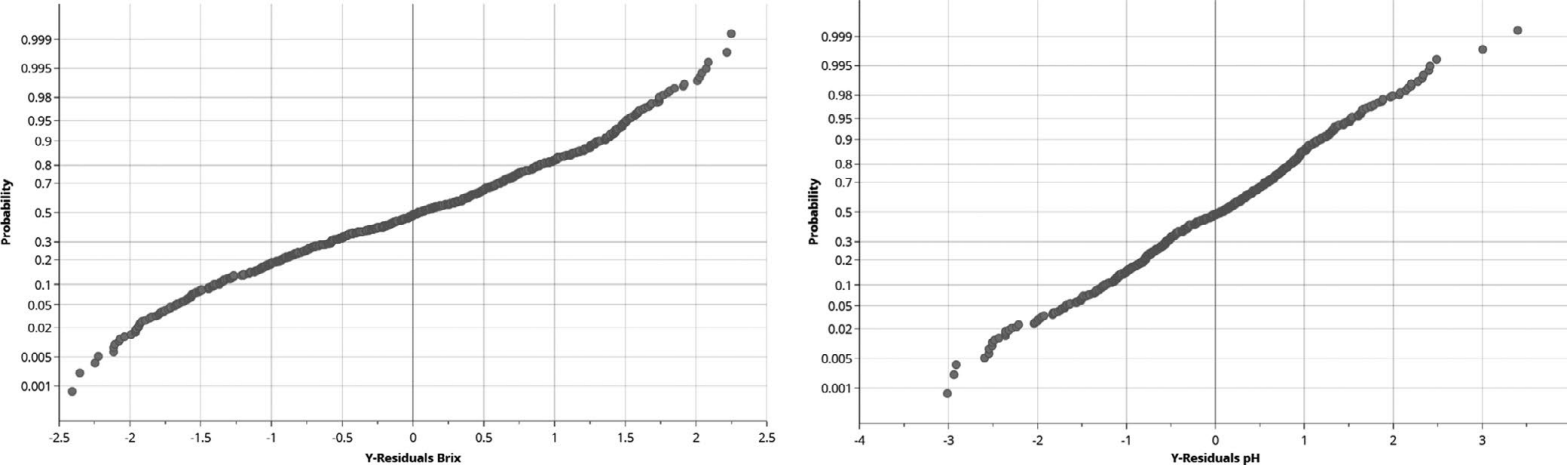
Residuals plots for the Brix and pH values of the PLSR model with a straight course representing normality

For the pursuing evaluation according to Figure 1, product properties of the juices were fitted with a PLSR model using the training set of fruit juice spectra. The optimization of the model quality was achieved by an iterative process with regard to the values of RMSEE and RMSECV. Included in the model were the analytical reference values of Brix and pH values, turbidity, and viscosity as Y variables. The number of latent variables was tested concerning overfitting by applying the permutation method. A summary of the PLSR model properties and quality parameters are shown in Table 3. RMSEE and RMSECV of the parameters Brix and pH values were very low in relation to the reference values and near 0, indicating the potential of these parameters as reference values. In order to verify the normality, the residual plots were considered. A linearity was found, and the limits of ±4 were not exceeded, which would be an indication of outlier. The residual plots of Brix and pH values were shown in Figure 6 as an example. In addition to examining the plots of residuals, an analysis of variance (ANOVA) was performed to test the significance of the models. The CV‐ANOVA provides a significance test of the null hypothesis of equal residuals of the two models compared. The *p* value is considered here, which indicates the probability level at which a model is recognized as significant, usually at a value of <.05. Since all the four Y variables resulted in *p* values smaller than 0.05, they can be described as significant. Results are shown in Table 8.

**TABLE 8:** Results of the ANOVA for the PLSR models of Brix and pH values, viscosity, and turbidity

Y variable of PLSR model	*F*	*P*
Brix	404.09	0
pH value	471.17	0
Viscosity	364.87	0
Turbidity	1108.56	0

**FIGURE 7:**
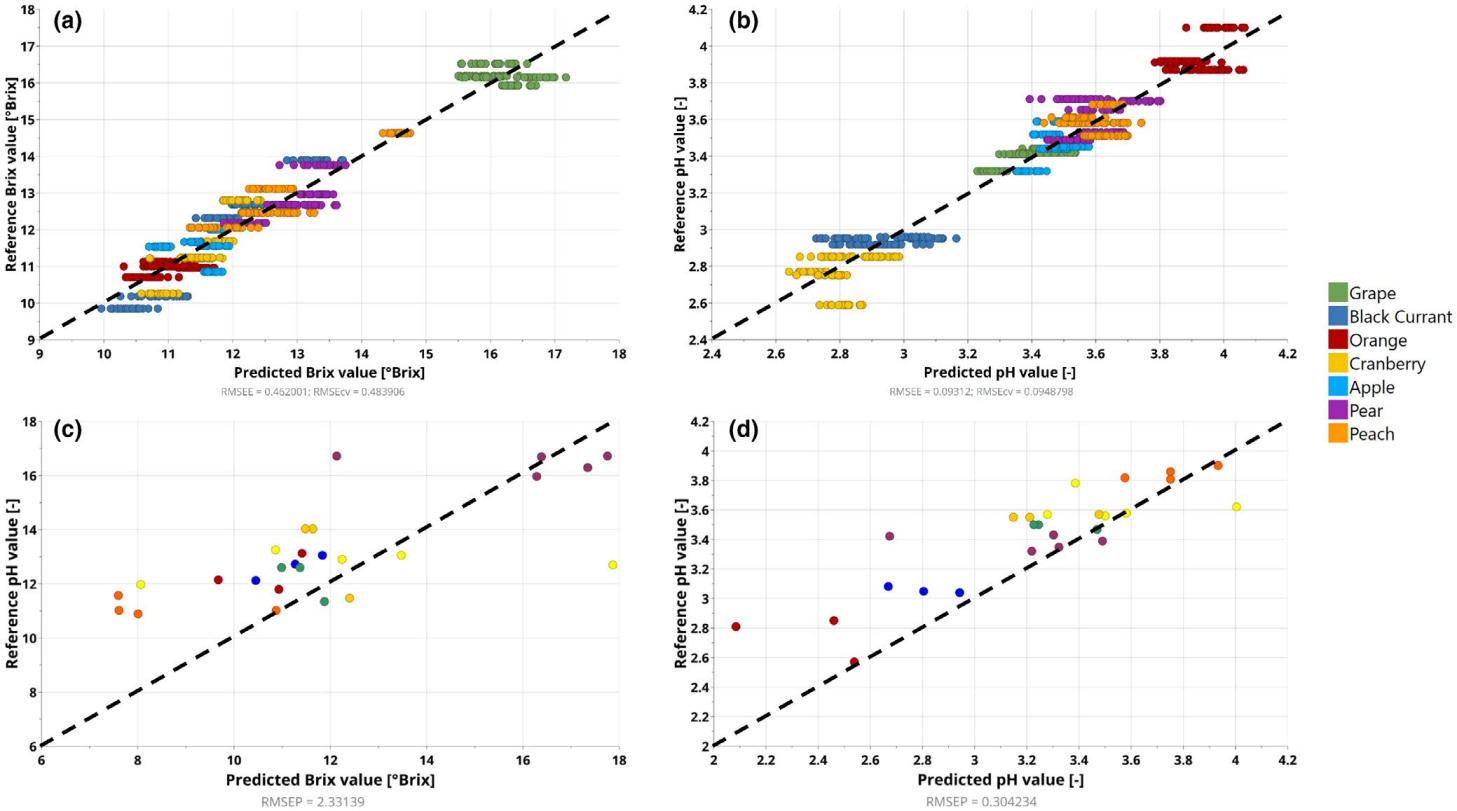
Scatter plots including predicted versus reference values regarding Brix (a) and pH (b) values for training set (RMSEE/ RMSEC) and external test set RMSEP (c, d)

For optimization and validation of prediction performance, the RMSEPs for the internal and external test sets were calculated (Table 9). RMSEP values for the internal test set are in the range of RMSEE and RMSECV (calculated with the training set), whereas the values of the external test set were only slightly higher in the case of the Brix and pH values (scatter plots are shown Figure 7). In case of viscosity and turbidity, an increase by a multiple of the RMSEP value was observed. A comparison of these results was shown in Table 10. The difference of the values with regard to the external test set shows that the model requires optimization with regard to the calibration dataset in order to increase the robustness against unknown samples. An increase in the number and diversity of the calibration dataset would provide this. In order to make the RMSEP values more interpretable, they were set in relation to the mean value of the reference values in order to determine the percentage error of prediction (Table 9). For the internal test set, percentage errors of <4% could be obtained for Brix and pH values, whereas for viscosity and turbidity this is above 15%. For the external test set, the percentage error values of the Brix value deteriorate to about 20%. With the external test set, higher prediction errors occur, which shows that the calibration dataset is too small. An increase of the sample size can lead to a significant improvement. Hence, it could be shown for the parameters Brix and pH values that they are suitable parameters for inline characterization by means of NIRS. For the parameters viscosity and turbidity, it is confirmed by a very strongly increased prediction error that they cannot be predicted well by NIRS. In conclusion and with the premise of model optimization, for example, in the form of increasing the dataset for decalibration, pH and Brix could be used as parameters for inline characterization of fruit juices by NIRS.

**TABLE 9:** Quality parameter RMSEP of PLSR model for the evaluation of prediction performance regarding the internal and external test sets and prediction error as relation between mean of reference values and RMSEP for a better interpretability

	RMSEP		Prediction error (%)	
Y variable	Internal test set	External test set	Internal test set	External test set
Brix value (°Brix)	0.48	2.33	3.83	18.58
pH value (–)	0.1	0.3	2.95	2.39
Turbidity (EBC)	77.19	940.34	18.58	7498.72
Viscosity (mPas)	1.39	3185.37	29.76	25401.67

**TABLE 10:** Quality parameter RMSEP of PLSR model in comparison to RMSEE and RMSECV for the evaluation of PLSR model performance

Y variable	RMSEE	RMSECV	RMSEP	
			Internal test set	External test set
Brix value (°Brix)	0.46	0.48	0.48	2.33
pH value (–)	0.09	0.09	0.1	0.3
Turbidity (EBC)	78.34	78.49	77.19	940.34
Viscosity (mPas)	1.36	1.45	1.39	3185.37

### 3.3 Exemplary application of an individualized pasteurization using product‐specific *D* and *z* values

Finally, an example is given how to profit in practice from an inline product identification and characterization using NIRS as analytic tool. A large number of *D* and *z* values are available and many of them are already collected in an Internet accessible database (“Lemgo D‐ and z‐value Database for Food”). However, more data are required for a wider use in practice. Despite its exemplary character, the case shown here is intended to demonstrate the feasibility of an individualized and gentle pasteurization by implementing NIRS as an inline analytical tool. A well‐known juice‐spoiling germ, *Alicyclobacillus acidoterrestris*, was therefore selected. In fruit juices, this organism can lead to product spoilage during warm storage (depending on climate and weather in certain countries), or hot packaging with slow cooling after too cold heat treatment, which stimulates the germination (Ciuffreda et al., 2015; Komitopoulou et al., 2001). The experience‐based recommendation for a sufficient treatment is 30 s at 101°C or expressed in pasteurization units 63 PU resulting from the so‐called fruit juice formula (ϑ_ref_ = 80°C, *z* = 10°C):

(1) $$PU=10^{\frac{\vartheta ‐\vartheta_{\mathrm{ref}}}{z}}\cdot t$$

In Equation 1, ϑ_ref_ corresponds to the reference temperature to which the *z* values used refer, ϑ corresponds to the actual temperature measured at the end of the heat‐holding section in classical control of the pasteurization process, and t represents the heating time.

In the here shown case, one (real) sample from the class apple and one from the category grape were randomly selected and characterized by PLSR method in Brix and pH values (shown in Table 11). Bibliographic data for the described characteristics of the different juice types are available, which show the influence of the product matrix on the heat stability of the microorganism *A*. *acidoterrestris* (Aneja et al., 2014; Silva et al., 1999). Silva et al. (1999) published a tabular overview of determined *D* and *z* values of different germs in different product matrices, among others also by Splittstoesser et al. (1994), who worked with well‐comparable juice in regard to pH value and Brix. Silva et al. (1999) elaborated an empirical model in terms of a polynomic function that allows the determination of the *D* value in dependence of the temperature, pH value, and extract content in Brix.

**TABLE 11:** Comparison of the required holding time in a flash pasteurizer at 95°C to apply a lethal impact of 63 PU based on experimental D/z values (Splittstoesser et al., 1994) on an empirical model (Silva et al., 1999) taking pH value and extract content

		Apple	Grape
(Selected samples) Inline NIR analysis/Predicted by PLSR model	pH value	3.5	3.3
	Extract in °Brix	11.9	16.3
Splittstoesser et al.	pH value	3.5	3.3
	Extract in °Brix	11.4	15.8
	*z* in °C	7.7	7.2
	Time required for 63 PU in seconds (95°C)	43	31
*D*/*Z* value model Calculated for the predicted pH and °Brix by Silva et al.^a^	*z* in °C	8.3	8.7
	Time required for 63 PU in seconds (95°C)	59	71
Conventional standard PU formula (“fruit juice formula”)	*z* in °C	10	10
	Time required for 63 PU in seconds (95°C)	120	120

a *z* value was calculated with the modeled D values at 80°C and 90°C for the predicted pH values and extract contents of the selected samples.

Two *D* values (for ϑ_1_ = 80°C and ϑ_2_ = 90°C) were calculated by the model of Silva et al. and from that the *z* values for the selected samples and the predicted pH values and extract contents:

(2) $$z=‐\frac{\vartheta_{1}‐\vartheta_{2}}{lg\frac{D_{\vartheta_{1}}}{D_{\vartheta_{2}}}}$$

In the formula, *lg* is an abbreviated notation of the logarithm of 10. For comparison purposes, the required holding times for 63 PU at 95°C were calculated, results are shown in Table 11. The “customization” with individual *z* values reveals that for the same PU impact a shorter treatment at 95°C seem to be sufficient in comparison to the fruit juice formula. However, the slight differences in pasteurization time do not allow a distinction to be made between grape and apple juices, whether calculated with *D* and *z* values according to Splittstoesser et al. or Silva et al. A more obvious difference could be expected in relation to cloudy juices or in comparison with beers depending on their cloudiness and alcohol and sugar content. What becomes already clear, however, is the big difference compared to the results of the conventional formula. Thus, it could be shown that an optimization of the pasteurization process can be realized by a more precise classification and characterization in the initial phase of the pasteurization by using NIRS as an inline analysis tool.

## 4 CONCLUSION

Using the PLS‐DA method, a classification model was created which is capable of distinguishing seven different fruit juices in the course of inline measurement using NIRS. In addition, these fruit juices were characterized in terms of microbiological properties by the two parameters Brix and pH values using the PLSR method. The turbidity and viscosity could not be represented by NIRS with high coefficients of determination, which led to regression models of low prediction quality. However, the extract content and the pH value are sufficient parameters for the practical application with regard to microbiological stability. The practical use of this information for process optimization was demonstrated using two examples. A clear difference between the two fruit juice examples was not observed, yet a strong difference with regard to the conventional method (“fruit juice formula”) was found. The applied strategy of inline analysis using NIRS and chemometrics is therefore suitable for product adaptation of the pasteurization process. However, it is questionable whether the ambitious plan to individualize pasteurization down to fine product properties can succeed. Therefore, it is necessary to further investigate how cloudy fruit juices differentiate from clear fruit juices or how this applies to different beer types. In conclusion, this study has provided a fundamental basis for further investigation to realize an individualized pasteurization.

## CONFLICT OF INTEREST

The authors declare no conflict of interest.

## AUTHOR CONTRIBUTIONS

**Imke Weishaupt:** Conceptualization (lead); Data curation (lead); Formal analysis (lead); Investigation (lead); Methodology (lead); Validation (lead); Writing – original draft (lead); Writing – review & editing (lead). **Peter Neubauer:** Conceptualization (supporting); Formal analysis (supporting); Methodology (supporting); Supervision (equal); Validation (supporting); Writing – review & editing (supporting). **Jan Schneider:** Conceptualization (supporting); Funding acquisition (lead); Methodology (supporting); Project administration (lead); Supervision (equal); Validation (supporting); Writing – review & editing (supporting).

## ETHICAL APPROVAL

The study does not involve any human or animal testing.

## Data Availability

Data are available upon request from the authors.
